# Supplementary material for: Oral angiotensin-converting enzyme inhibitor captopril protects the heart from Porphyromonas gingivalis LPS-induced cardiac dysfunction in mice
Source: PLoS One. 2023 Nov 20;18(11):e0292624. doi: 10.1371/journal.pone.0292624 (PMC10659197; doi:10.1371/journal.pone.0292624)
Supplement: S1 Data — (PDF) [file pone.0292624.s001.pdf]

S1 Data

Oral angiotensin-converting enzyme inhibitor captopril protects the heart from *Porphyromonas gingivalis* LPS-induced cardiac dysfunction in mice

Running title: RAS activation in the heart of periodontitis

Kenichi Kiyomoto [1,2 ¶], Ichiro Matsuo [2 ¶], Kenji Suita [1], Yoshiki Ohnuki [1], Misao Ishikawa [3], Aiko Ito [4], Yasumasa Mototani [1], Michinori Tsunoda [1,2], Akinaka Morii [1,2], Megumi Nariyama [5], Yoshio Hayakawa [6], Yasuharu Amitani [7], Kazuhiro Gomi [2], Satoshi Okumura [1]

[1] Department of Physiology, Tsurumi University School of Dental Medicine, Yokohama 230-8501, Japan

[2] Department of Periodontology, Tsurumi University School of Dental Medicine, Yokohama 230-8501, Japan

[3] Department of Oral Anatomy, Tsurumi University School of Dental Medicine, Yokohama 230-8501, Japan

[4] Department of Orthodontology, Tsurumi University School of Dental Medicine,

Yokohama 230-8501, Japan

[5] Department of Pediatric Dentistry, Tsurumi University School of Dental Medicine,

Yokohama 236-8501, Japan

[6] Department of Dental Anesthesiology, Tsurumi University School of Dental Medicine,

Yokohama 230-8501, Japan

[7] Department of Mathematics, Tsurumi University School of Dental Medicine,

Yokohama, Japan

¶ These authors contributed equally to this work.

*****Corresponding author: Satoshi Okumura:

Department of Physiology, Tsurumi University School of Dental Medicine,

2-1-3 Tsurumi, Tsurumi-ku, Yokohama 230-8501; (Tel. +81-(0)45-580-8476;

Fax. +81-(0)45-585-2889; e-mail: okumura-s@tsurumi-u.ac.jp)

Supplemental Figure 1

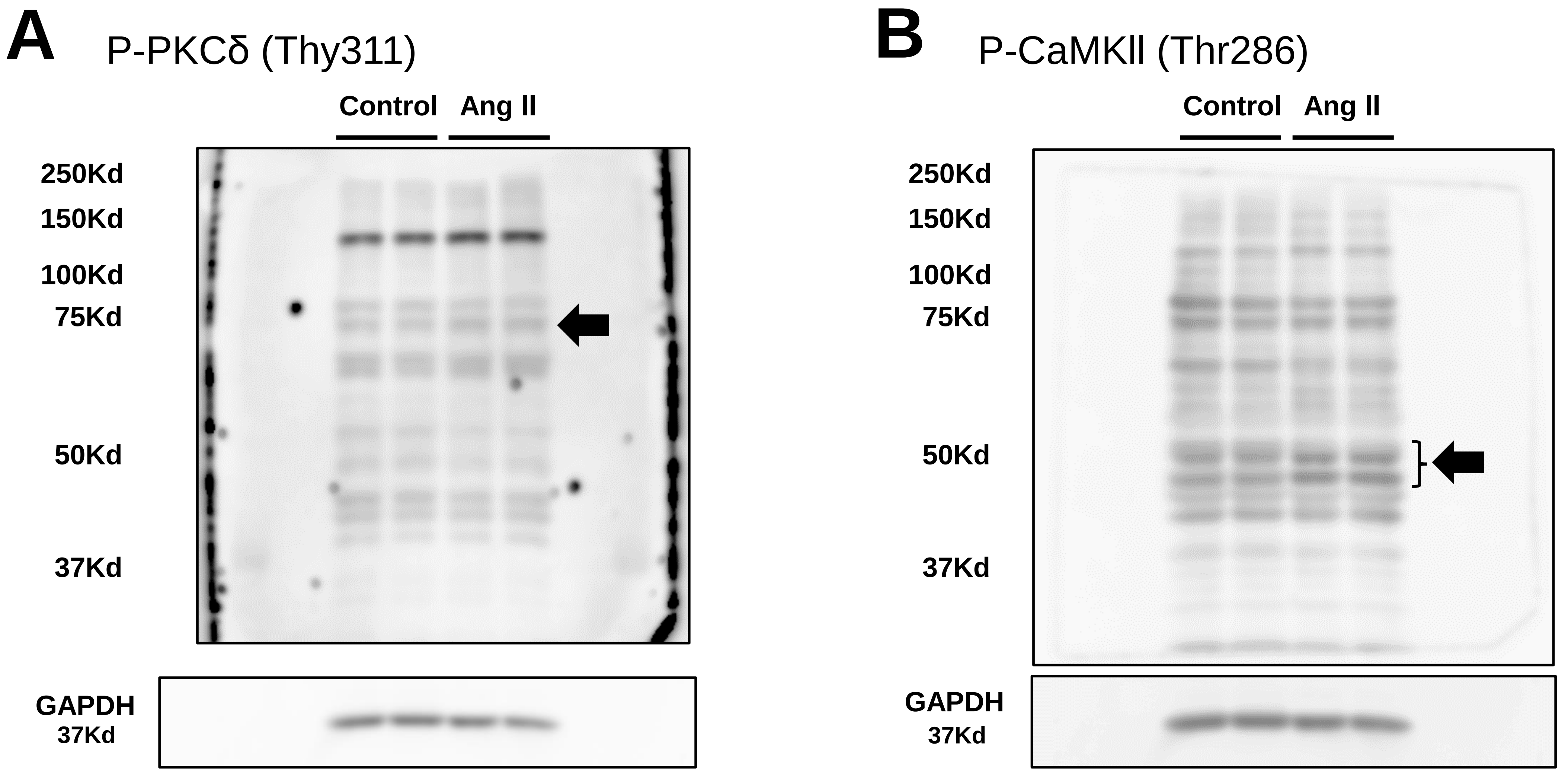

Supplemental Figure 1.
Immonoblots of phospho-PKCδ (Thr311) (A) and CaMKII (Thr286) (B) in cardiac tissue homogenate are cardiac tissue homogenate prepared from control (left) and Ang II-treated mice (right). The density of the bands indicated by the arrows was increased by the Ang II treatment.

Supplemental Figure 2

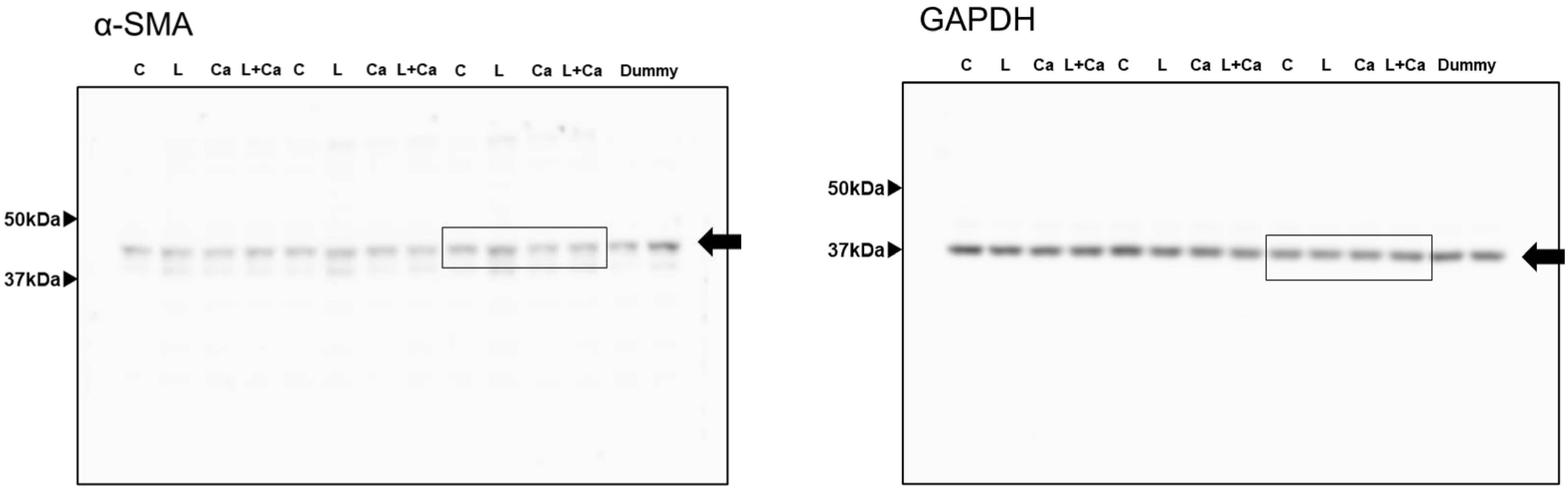

Supplemental Figure 2.
Representative full-length immunoblots of Fig. 3C. The amount of α-SMA and GAPDH were shown.
The black-line box indicated by arrow in each blot is corresponded to the cropped parts that are showed in the main article.

Supplemental Figure 3

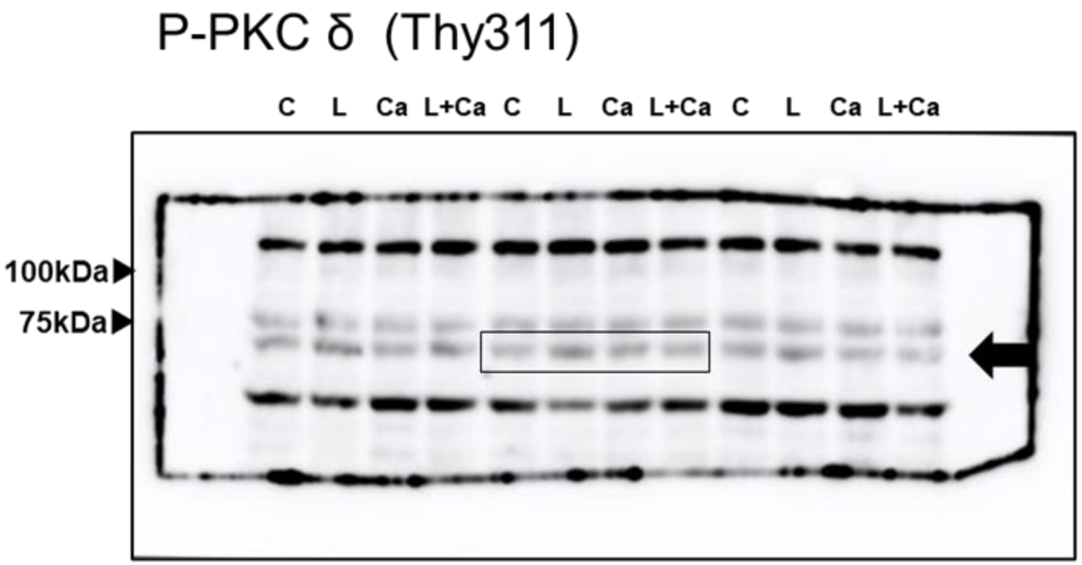

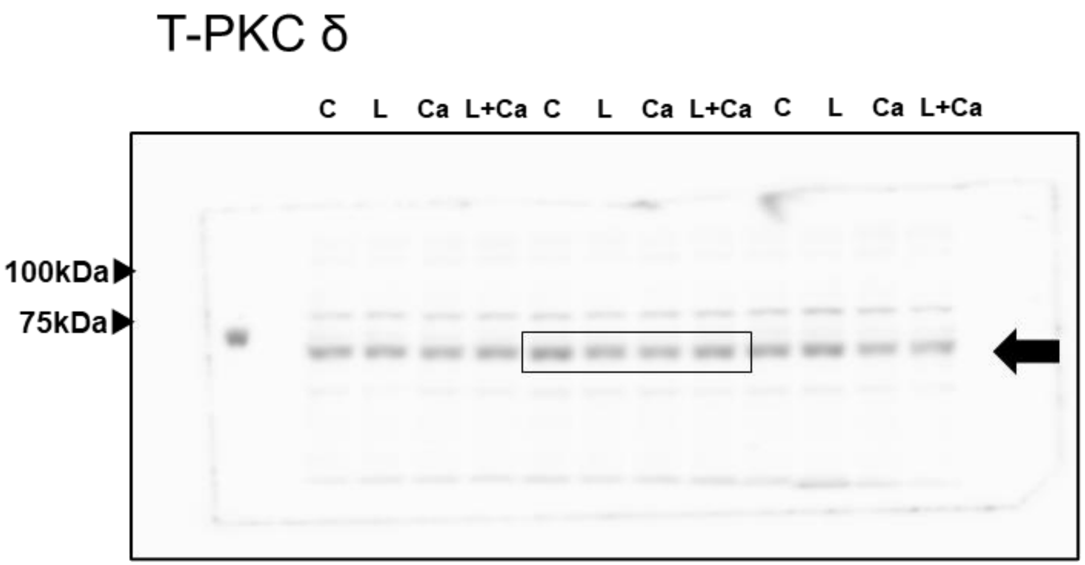

Supplemental Figure 3.
Representative full-length immunoblots of Fig. 5A. The amount of P-PKCδ (Thy311) and T-PKCδ were shown.
The black-line box indicated by arrow in each blot is corresponded to the cropped parts that are showed in the main article.

Supplemental Figure 4

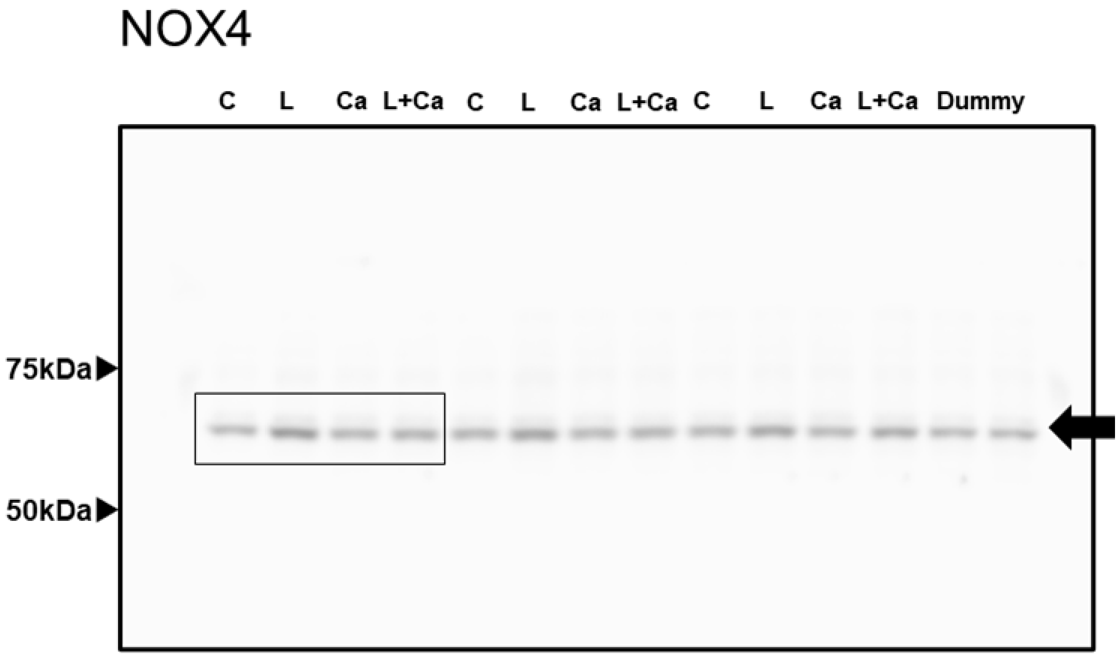

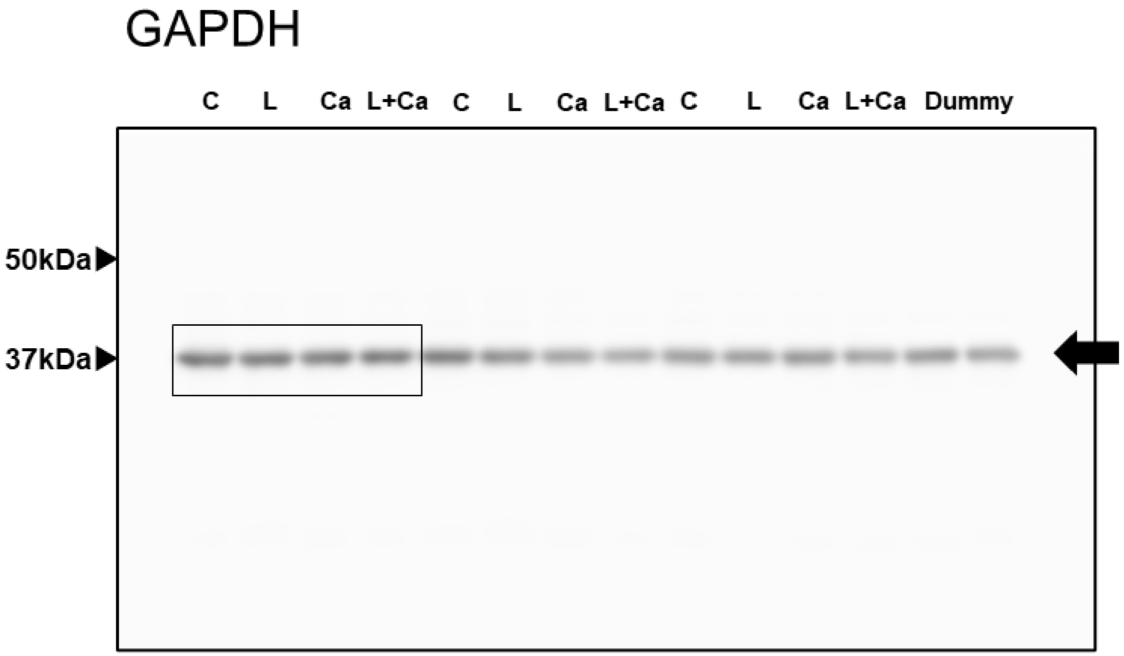

Supplemental Figure 4.
Representative full-length immunoblots of Fig. 5B. The amount of NOX4 and GAPDH were shown.
The black-line box indicated by arrow in each blot is corresponded to the cropped parts that are showed in the main article.

Supplemental Figure 5

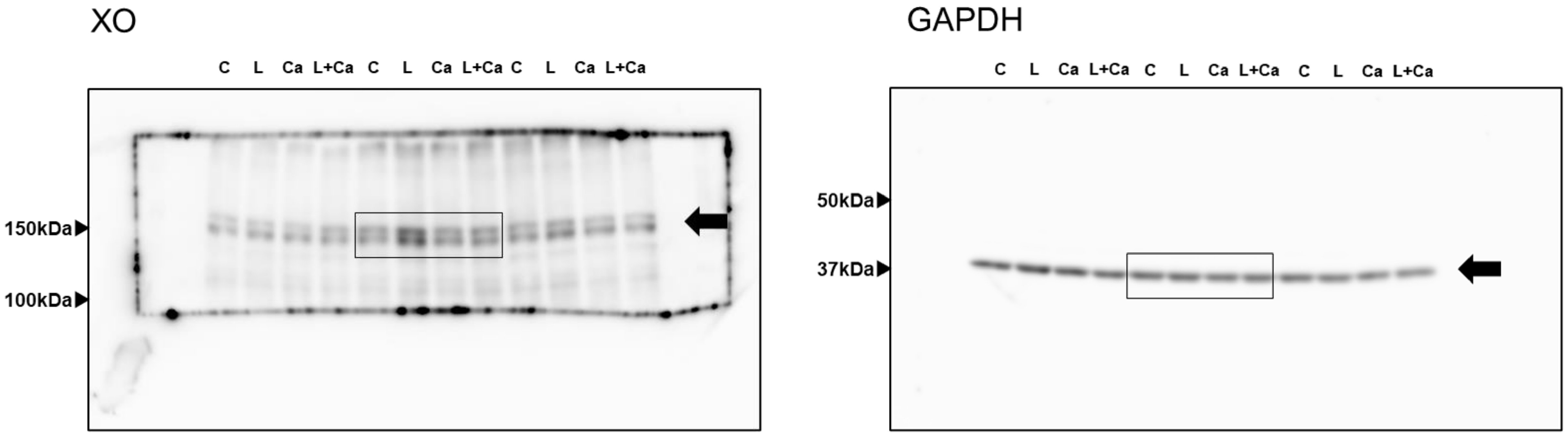

Supplemental Figure 5.
Representative full-length immunoblots of Fig. 5C. The amount of Xanthine oxidase and GAPDH were shown.
The black-line box indicated by arrow in each blot is corresponded to the cropped parts that are showed in the main article.

Supplemental Figure 6

P-CaMKII (Thr286)

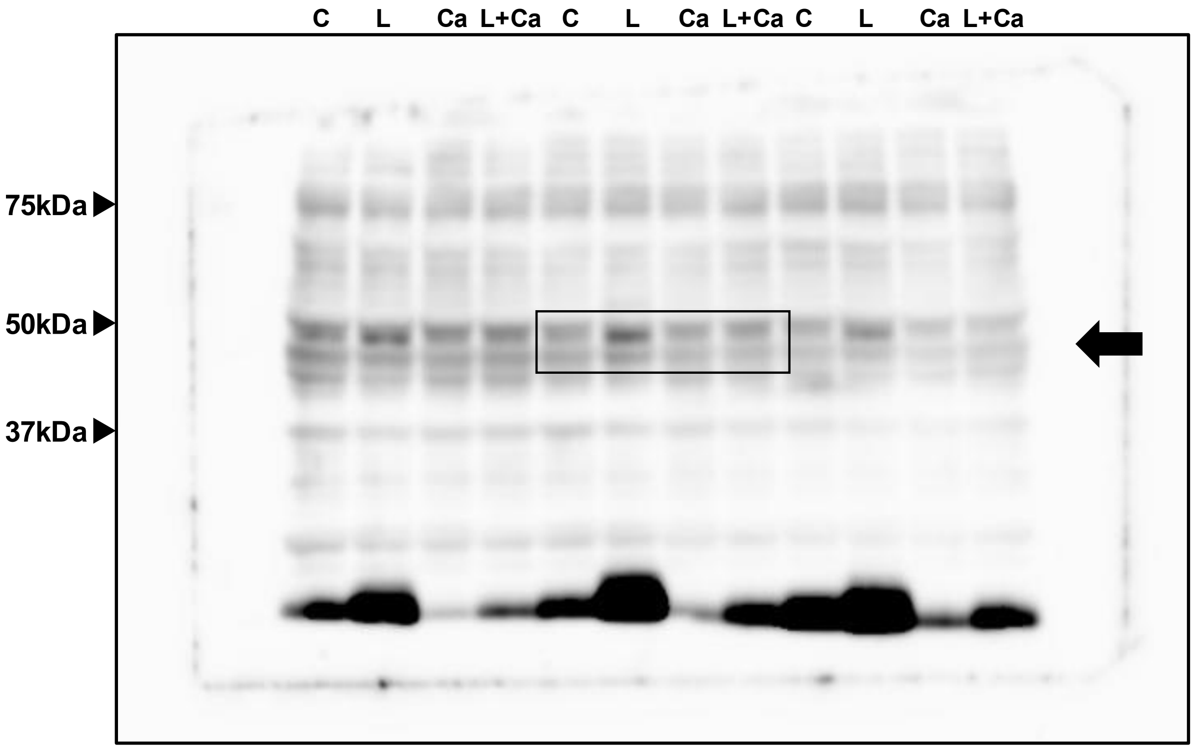

T-CaMKII

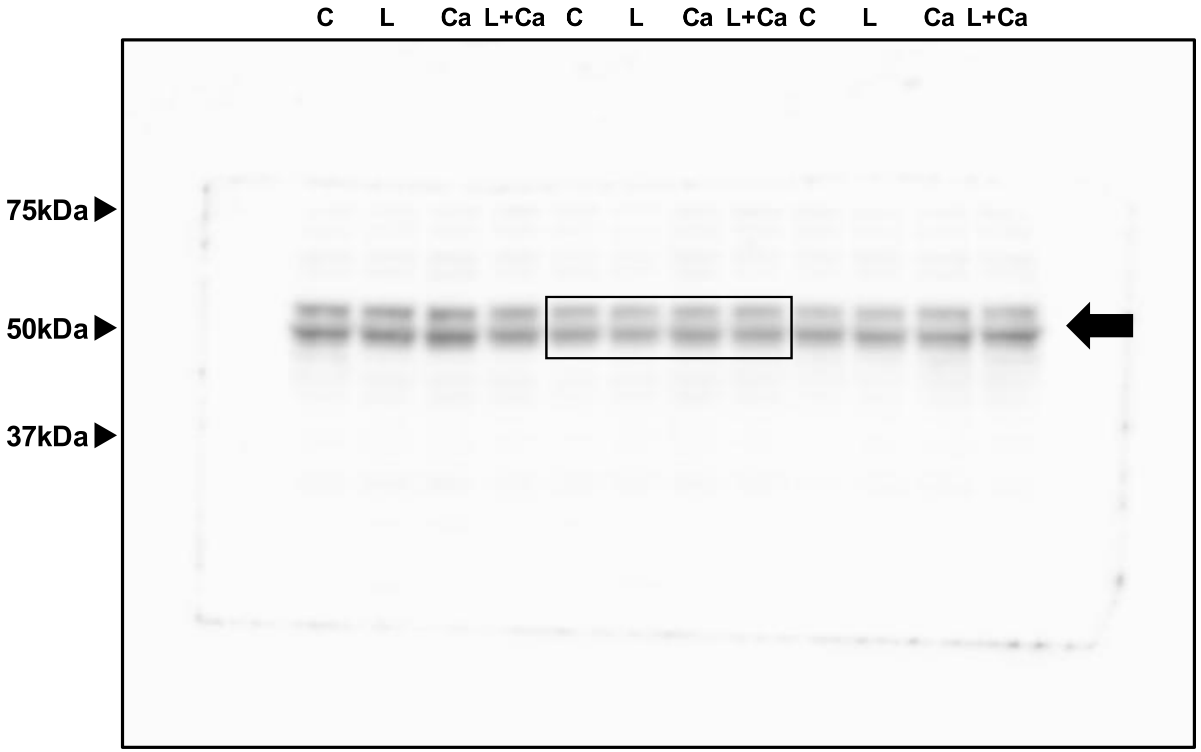

Supplemental Figure 6.
Representative full-length immunoblots of Fig. 5D. The amount of P-CaMKII (Thr286) and T-CaMKII were shown.
The black-line box indicated by arrow in each blot is corresponded to the cropped parts that are showed in the main article.

Supplemental Figure 7

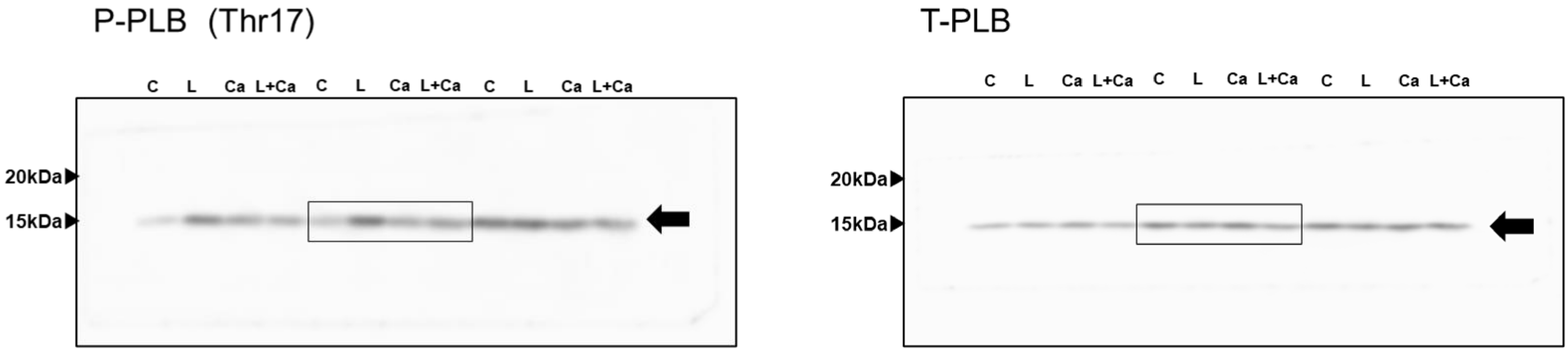

Supplemental Figure 7.
Representative full-length immunoblots of Fig.5E. The amount of P-PLB(Thr17) and T-PLB were shown.
The black-line box indicated by arrow in each blot is corresponded to the cropped parts that are showed in the main article.
