# Supplementary material for: Oral angiotensin-converting enzyme inhibitor captopril protects the heart from Porphyromonas gingivalis LPS-induced cardiac dysfunction in mice
Source: PLoS One. 2023 Nov 20;18(11):e0292624. doi: 10.1371/journal.pone.0292624 (PMC10659197; doi:10.1371/journal.pone.0292624)
Supplement: S2 Data — (PDF) [file pone.0292624.s002.pdf]

S2 Data

Oral angiotensin-converting enzyme inhibitor captopril protects the heart from *Porphyromonas gingivalis* LPS-induced cardiac dysfunction in mice

Running title: RAS activation in the heart of periodontitis

Kenichi Kiyomoto [1,2 ¶], Ichiro Matsuo [2 ¶], Kenji Suita [1], Yoshiki Ohnuki [1], Misao Ishikawa [3], Aiko Ito [4], Yasumasa Mototani [1], Michinori Tsunoda [1,2], Akinaka Morii [1,2], Megumi Nariyama [5], Yoshio Hayakawa [6], Yasuharu Amitani [7], Kazuhiro Gomi [2], Satoshi Okumura [1]

[1] Department of Physiology, Tsurumi University School of Dental Medicine, Yokohama 230-8501, Japan

[2] Department of Periodontology, Tsurumi University School of Dental Medicine, Yokohama 230-8501, Japan

[3] Department of Oral Anatomy, Tsurumi University School of Dental Medicine, Yokohama 230-8501, Japan

[4] Department of Orthodontology, Tsurumi University School of Dental Medicine,

Yokohama 230-8501, Japan

[5] Department of Pediatric Dentistry, Tsurumi University School of Dental Medicine, Yokohama 236-8501, Japan

[6] Department of Dental Anesthesiology, Tsurumi University School of Dental Medicine, Yokohama 230-8501, Japan

[7] Department of Mathematics, Tsurumi University School of Dental Medicine, Yokohama, Japan

¶ These authors contributed equally to this work.

*****Corresponding author: Satoshi Okumura:

Department of Physiology, Tsurumi University School of Dental Medicine,

2-1-3 Tsurumi, Tsurumi-ku, Yokohama 230-8501; (Tel. +81-(0)45-580-8476;

Fax. +81-(0)45-585-2889; e-mail: okumura-s@tsurumi-u.ac.jp)

Tables of Effect Sizes, Sample Sizes, and Statistical Powers

In what follows, the symbol (*) stands for statistically significant at the level α = 0.05.

◆ Fig.1（1-way ANOVA）

		**A priori（α=0.05, 1-β=0.80）**		**Post hoc**				**A posteriori (α=0.05, 1-β=0.80)**
		Total sample size	Effect size *f*	*p* -value	Power	Actual total sample size (α=0.05)	Effect size *f*	Total sample size
B	Food	76	0.4	0.170809039	0.3720141	25	0.4470368	60
B	Water	76	0.4	0.048381406	0.5281754	25	0.5432426	44
C	Ang ll*	76	0.4	0.012806988	0.5592543	17	0.7209491	28

◆ Fig.2（1-way ANOVA）

		**A priori（α=0.05, 1-β=0.80）**		**Post hoc**				**A posteriori（α=0.05, 1-β=0.80）**
		Total sample size	Effect size *f*	*p* -value	Power	Actual total sample size (α=0.05)	Effect size *f*	Total sample size
A	Body weight	76	0.4	0.723343507	0.1306128	26	0.2345063	204
B	CM/TL	76	0.4	0.713399997	0.1333695	26	0.2380957	200
C	Lung	76	0.4	0.75208014	0.1227675	26	0.223874	224
D	Liver	76	0.4	0.332785613	0.2710328	26	0.3677816	88

◆ Fig.3（1-way ANOVA）

		**A priori（α=0.05, 1-β=0.80）**		**Post hoc**				**A posteriori（α=0.05, 1-β=0.80）**
		Total sample size	Effect size *f*	*p* -value	Power	Actual total sample size (α=0.05)	Effect size *f*	Total sample size
B	Fibrosis area*	76	0.4	2.28718E-07	0.9373	26	0.8646	20
C	α-SMA/GAPDH*	76	0.4	0.00063034	0.7447	20	0.7855	24

◆ Fig.4（1-way ANOVA）

		**A priori（α=0.05, 1-β=0.80）**		**Post hoc**				**A posteriori（α=0.05, 1-β=0.80）**
		Total sample size	Effect size *f*	*p* -value	Power	Actual total sample size (α=0.05)	Effect size *f*	Total sample size
B	TUNEL positive*	76	0.4	2.24795E-05	0.7258566	16	0.8968982	20
C	BCL-2/GAPDH*	76	0.4	9.81E-05	0.7757623	19	0.8406886	20

◆ Fig.5（1-way ANOVA）

		**A priori（α=0.05, 1-β=0.80）**		**Post hoc**				**A posteriori（α=0.05, 1-β=0.80）**
		Total sample size	Effect size *f*	*p* -value	Power	Actual total sample size (α=0.05)	Effect size *f*	Total sample size
A	AT1-AR/GAPDH	76	0.4	0.372072892	0.252228	25	0.3534624	92
B	P-PKCδ(Tyr311)/PKCδ*	76	0.4	0.001385079	0.7167562	20	0.7625465	24
C	NOX4/GAPDH*	76	0.4	0.003676402	0.7088955	23	0.6917034	28
D	XO/GAPDH*	76	0.4	2.151E-05	0.883366	25	0.8068684	24
E	P-CaMKll(Thr286)/T-CaMKll*	76	0.4	0.000970177	0.730035	20	0.7733274	24
F	P-PLB(Thr17)/T-PLB*	76	0.4	0.00316393	0.6943468	21	0.7216932	28

◆ Table.1（1-way ANOVA）

		**A priori（α=0.05, 1-β=0.80）**		**Post hoc**				**A posteriori（α=0.05, 1-β=0.80）**
		Total sample size	Effect size *f*	*p* -value	Power	Actual total sample size (α=0.05)	Effect size *f*	Total sample size
A	EF*	76	0.4	0.000111542	0.8521397	25	0.7728599	24
B	EDV	76	0.4	0.555566183	0.1803419	25	0.2977543	128
C	ESV	76	0.4	0.080835261	0.4703095	25	0.5083481	48
D	FS*	76	0.4	0.000118063	0.8508341	25	0.7715482	24
E	LVIDd	76	0.4	0.582748967	0.1715354	25	0.2886284	136
F	LVIDs	76	0.4	0.089325173	0.4581732	25	0.500971	48
G	HR	76	0.4	0.702851308	0.1358759	25	0.2470489	184
H	SV	76	0.4	0.080835261	0.4703095	25	0.5083481	48
I	CO	76	0.4	0.063509127	0.4984281	25	0.525339	44
J	IVSTd	76	0.4	0.395968863	0.2402016	25	0.3521664	92
K	LVSTs	76	0.4	0.11066499	0.4311336	25	0.4843891	52
L	LVPWTd	76	0.4	0.299846002	0.2863614	25	0.3880835	80
M	LVPWTs	76	0.4	0.024009096	0.5959378	25	0.5843131	40
