# Supplementary material for: Oral angiotensin-converting enzyme inhibitor captopril protects the heart from Porphyromonas gingivalis LPS-induced cardiac dysfunction in mice
Source: PLoS One. 2023 Nov 20;18(11):e0292624. doi: 10.1371/journal.pone.0292624 (PMC10659197; doi:10.1371/journal.pone.0292624)
Supplement: Attachment — Submitted filename: PLOS ONE Response revised.pdf [file pone.0292624.s003.pdf]

Reviewer #1:

The present study aimed to evaluate the possible protective effects of oral Captopril in mice heart infected with Porphylomonas gingivalis LPS (LPS-Pg), i.p. The relationship between poor oral health and cardiovascular complications, such as heart failure and fibrosis, is widespread in the literature. The important role of RAS components in cardiac functions and structures is also known, especially when it comes to Ang-II peptide linked to AT1 receptor.

Criticism-1:

It was the hypothesis of this study that there would be cardiovascular protection promoted by Captopril with regard to decrease in cardiac fibrosis caused by LPS-Pg, as well as an improvement in ejection fraction also in mice treated with LPS-Pg. However, the sample size calculation for carrying out this study was not presented, so there is no guarantee that the number of mice used reflect the reality of the results.

Response-1-(1): We asked Dr. Yasuharu Amitani, a statistician, who is included as a co-author in the revised manuscript, to examine the statistical validity in response to the comments of Reviewers #1 and #3. We calculated the total sample size of animals (α risk = 0.05, power ($1-\beta$) = 0.8) not only *a priori* (effect size (f) = 0.4) but also *a posteriori* with the effects size (f) derived *post hoc* [1] by means of G^* Power version 3.1 (program, concept and design by Franz, Universitat Kiel, Germany; freely available Windows application software) [2] (**S2 Data)**. In regard to the data with the statistically significant differences: Fig 1C, 3B, 3C, 4B, 4C, 5B-F and Table 1A (EF) and 1D (%FS)

(**S2 Data)**, the actual sample sizes are close to the required sample sizes (*n* = 20 - 28) because the a *posteriori* effect sizes (*f*) are much greater than the *a priori* size (*f* = 0.4) [1]. Therefore, the sample size used in this study might be sufficient to evaluate the significant differences.

We incorporated the following sentences in the method section of the revised manuscript (Page 13, Lines 4-8).

We calculated the required total sample size of animals (α risk = 0.05, power (1-β) = 0.8) not only *a priori* (effect size (*f*) = 0.4) but also *a posterior* with the effects size (*f*) derived from *post hoc* [1] by means of G* Power version 3.1 (program, concept and design by Franz, Universitat Kiel, Germany; freely available Windows application software) [2] (**S2 Data)**.

Response-1-(2): However, in regard to the data without statistically significant differences: Fig 1B, 2A-D, 5A and Table 1B, 1C, 1E-M), the actual sample sizes are small, compared to the required sample size *a posteriori* (40-224). Although it is not possible to rule out the possibility that the lack of a detected difference is due to the relatively small sample size, we could not prepare enough animals within a reasonable timeframe. There are also ethical issues associated with the use of large number of animals.

Instead, we have incorporated a comment on this issue as a study limitation in the results section of the revised manuscript as shown below.

1) Page 14, Lines 13-17

---chronic PG-LPS treatment with or without Cap under the experimental conditions used in this study had no significant effect on growth, food consumption or water consumption, although we cannot rule out the possibility that the apparent lack of difference was due to the relatively small number of animals used.

2) Page 18, Lines 11-15

We thus examined the expression of AT1 receptors in the heart and found that the levels were similar among the four groups, although we cannot rule out the possibility that the apparent lack of difference was due to the relatively small number of animals used **(Fig 5A)**.

Criticism-2:

In addition, the figure 6 concludes that the increase in oxidative stress with consequent increase in apoptosis and cardiac fibrosis, as well as the left ventricular ejection fraction worsening, is linked to Ang-II and AT1 receptors interaction. Bearing this in mind, the Captopril use could decrease the Ang-II availability and contribute to the improvement of cardiac function in mice infected with LPS-Pg. On the other hand, there is no mention in this study other possibilities of Ang-II formation by other enzymatic pathways besides the Converse Angiotensin enzyme (ACE) function. Elastase-2, for example, can cleave angiotensinogen directly into Ang-II, so an Ang-II dosage throughout the study is essential for figure 6 conclusion.

<u>Response-2</u>:

We examined serum levels of Ang II by means of ELISA. Serum Ang II levels were significantly increased in the PG-LPS group compared to the control (Control [*n* = 4] vs. PG-LPS [*n* = 4]: 88 ± 12 vs. 759 ± 624, $P < 0.05$ by one-way ANOVA followed by the Tukey-Kramer *post hoc* test), and the increase was significantly suppressed by Cap (PG-LPS [*n* = 4] vs. PG-LPS [*n* = 5]: 759 ± 624 vs. 62 ± 67, $P < 0.05$ by one-way ANOVA followed by the Tukey-Kramer *post hoc* test **(Fig 1C)**.

These results suggest that activation of RAS might be mediated by PG-LPS treatment.

We incorporated the above sentences in the result section of the revised manuscript as shown below (Page 15, Line 12-Page 16, Line 3).

Effects of PG-LPS on serum Ang II levels

The renin-angiotensin system is thought to be involved in inflammatory processes such as periodontitis. However, its precise role is still unclear. Therefore, we next examined serum levels of Ang II in the four groups by means of ELISA. Serum Ang II levels were significantly increased in the PG-LPS group compared to the control (Control [*n* = 4] vs. PG-LPS [*n* = 4]: 88 ± 12 vs. 759 ± 624 pg/ml, $P < 0.05$ by one-way ANOVA followed by the Tukey-Kramer *post hoc* test), and the increase was significantly suppressed by Cap (PG-LPS [*n* = 4] vs. PG-LPS + Cap [*n* = 5]: 759 ± 624 vs. 62 ± 67 pg/ml, $P < 0.05$ by one-way ANOVA followed by the Tukey-Kramer *post hoc* test **(Fig 1C)**.

These results suggest that activation of RAS might be mediated by PG-LPS

treatment.

<u>Criticism-3:</u>

Finally, it would be interesting and important to have access to complete Western Blot membranes photos of all target studies because in some cases, such as the making for BCL-2, AT-1 and XO, the bands shown are double or triple and this can configure low antibodies selectivity and efficiency. It is worth mentioning that the presented images blots are cropped.

<u>Response-3</u>:

We performed Western blotting of BCL-2, AT-1 and XO using immunoreaction enhancer solution (Can Get Signal; TOYOBO, #NKB-101, Osaka, Japan) to improve the sensitivity and specificity. We replaced the blots of BCL-2, AT-1 and XO with the new blots in the revised manuscript, and show the complete blots as supplementary data **(see Fig 4C, Fig 5A, Fig 5D, S2 Fig of S1 Data, S3 Fig of S1 Data, and S6 Fig of S1 Data in the revised manuscript)**.

We modified the sentences in the method section of the revised manuscript as shown below (Page 12, Lines 9-11).

The primary and secondary antibodies were diluted in Tris-buffered saline (pH 7.6) with 0.1% Tween 20 and 5% bovine serum albumin or immunoreaction enhancer solution (Can Get Signal; TOYOBO, #NKB-101, Osaka, Japan).

Reviewer #2

The goal of this study was to investigate the effects of the ACE inhibitor captopril on Porphyromonas gingivalis lipopolysaccharide (PG-LPS) induced cardiac dysfunction in mice. The finding of this study are interesting and align with effects of oral disease state in the heart however, the conclusion that RAS is involved is a jump that is not substantiated by the data. In addition, there is clear issue with the data presented specifically with the echo and immunoblotting.

Criticism-1:

Immunoblots are not convincing as they are highly zoomed in and blurry. In some cases, the images do not visually look different despite the graph demonstrating a 6x difference. The images in the supplement are also highly overexposed for some of the blots. How do you know that these bands are specific?

Response-1:

The reviewer may be referring especially to the Western blot data of XO **(Fig 5D)**, i.e., the image does not visually look different despite the graph demonstrating a 6x difference. Please see our responses to the criticism-3 from the reviewer #1 and the criticism-2 from the reviewer #3. We have replaced the blots of XO with BCL-2 and AT-1 with new blots obtained using immunoreaction enhancer solution in the revised manuscript **(see Fig 4C, Fig 5A, Fig 5D, S2 Fig of S1 Data, S3 Fig of S1 Data, and S6 Fig of S1 Data in the revised manuscript)**

Criticism-2:

Some of the echo parameters are off such as cardiac output which is double what is expected in a mouse and wall thickness are significantly smaller. EDV and ESV also seem very small. It is not clear if these measurements were taken from both long and short axis measurements at midpapillary?

Response-2:

All LV dimensions are presented as the average of four consecutive selected beats. Heart rate (HR) was determined from the cardiac cycles recorded on the M-mode tracing, using at least three consecutive beats. The other parameters were calculated from M-mode-derived LV dimensions using the Teichholz formula [3]:

●EDV = (7 x $LVIDd^3$/1000) / (2.4 + (LVIDd/10)) and ESV = (7 x $LVIDs^3$/1000) / (2.4 + (LVIDd/10)) (mL)

EDV (mL): left ventricular end-diastolic volume

ESV (mL): left ventricular end-systolic volume

LVIDd (mm): left ventricular internal dimension at end-diastole

LVIDs (mm): left ventricular internal dimension at end-systole

● Stroke volume (SV) = EDV- ESV (mL)

● Cardiac output (CO) = HR x SV (ml/min)

● left ventricular ejection fraction (EF) = 100 x SV / EDV (%)

● left ventricular fractional shortening (%FS) = 100 x (LVIDd - LVIDs) / LVIDd (%)

All LV dimensions calculated using Teichholz formula in wild-type control (12-week-old C57BL/6 mice) shown in this study were consistent with those reported in previous studies by us [4] and another group [5].

We incorporated the above comments in the method section of the revised manuscript (Page 9, Lines 1-17).

Criticism-3:

It is unclear how the conclusion was mad that PGLPS was increasing RAS when these was no change in the AT receptor. While the downstream intracellular mechanism make sense, there is a clear connection to the RAS system. It is possible that activation of RAS is not directly through Pg-LPS but another indirect pathway.

Response-2:

Please see our responses to the criticism-2 from reviewer #1.

Minor:

Criticism-1:

Grammatic issue with sentences in abstract 3rd line: 'have shown little beneficial effects of ACE inhibitors in animals with poor oral health, particularly periodontitis, has been shown in some experimental failing hearts' which has two verbs.

Response-1:

Thank you. We have corrected it as shown below (Page 3, Lines 4-5).

---have shown little beneficial effects of ACE inhibitors in animals with oral health, particular periodontitis. In this study, we examined---

<u>Crtiticism-2</u>:

Page 7 in methods that is duplicated on last line.

<u>Response-2</u>:

Thank you. We corrected it as shown below (Page 7, Line 17-Page 8, Line 1)..

---this study is consistent with the circulating levels in patients with periodontitis, so that this model is not a sepsis model---

Reviewer 3:

It is well-structured manuscript with great scientific and clinical effects, which is why I suggest its publication, however the following adjustments are required.

<u>Crtiticism-1</u>:

In the methodology, mention how the sample size of n: 6 of the animals per treatment group is determined.

Response-1:

Please see our responses to the criticism-1 from reviewer #1.

Criticism-2:

Review the images of the western blot results, seven drafts of the bands and in some cases the relationship of those described and observed in the figure of bars with the increase in expression is not seen.

Response-2:

We think the reviewer was referring the Western blots of PKC-δ and XO **(Fig 5B and D)**. Please see our responses to the criticism-3 from the reviewer #1 and criticism-1 from the reviewer #2. We replaced the blots of PKC-δ and XO with BCL-2 and AT-1 with new blots obtained using immunoreaction enhancer solution in the revised manuscript **(see Fig 4C, Fig 5A, Fig 5B, Fig 5D, S2 Fig of S1 Data, S3 Fig of S1 data, S4 Fig of S1 Data and S6 Fig of S1 data in the revised manuscript)**.

Criticism-3:

In the discussion they focus on describing the results obtained in this manuscript with what was previously obtained by the same authors, it is necessary to seek other scientific contributions described by other researchers.

Response-3:

The involvement of the RAS in myocardial fibrosis is evident in several pathological conditions such as hypertensive heart disease [6], congestive heart failure [7] and myocardial infarction [8]. It has been suggested that PD may be a contributory risk factor for CVD [9]. Although many studies have found an association between PD and CVD, the relationship is still controversial and is thought to require more

investigation [10, 11]. To our knowledge, this study is the first to establish an association between PD and myocardial fibrosis and to afford evidence that RAS might be a key mediator for this association.

We incorporated the above sentences in the discussion portion of the revised manuscript (Page 27, Lines 1-8).

References

1. Cohen J. A power primer. Psychol Bull. 1992;112(1):155-9.
2. Faul F, Erdfelder E, Buchner A, Lang AG. Statistical power analyses using G*Power 3.1: tests for correlation and regression analyses. Behav Res Methods. 2009;41(4):1149-60.
3. Teichholz LE, Kreulen T, Herman MV, Gorlin R. Problems in echocardiographic volume determinations: echocardiographic-angiographic correlations in the presence of absence of asynergy. Am J Cardiol. 1976;37(1):7-11.
4. Matsuo I, Kawamura N, Ohnuki Y, Suita K, Ishikawa M, Matsubara T, et al. Role of TLR4 signaling on Porphyromonas gingivalis LPS-induced cardiac dysfunction in mice. PLoS One. 2022;17(6):e0258823.
5. Jones AA, Framnes-DeBoer SN, Shipp A, Arble DM. Caloric restriction prevents obesity- and intermittent hypoxia-induced cardiac remodeling in leptin-deficient ob/ob mice. Front Physiol. 2022;13:963762.
6. Brilla CG, Pick R, Tan LB, Janicki JS, Weber KT. Remodeling of the rat right and left ventricles in experimental hypertension. Circ Res. 1990;67(6):1355-64.
7. Weber KT, Brilla CG, Janicki JS. Myocardial fibrosis: functional significance and regulatory factors. Cardiovasc Res. 1993;27(3):341-8. Epub 1993/03/01. doi: 10.1093/cvr/27.3.341. PubMed PMID: 8490934.
8. Hanatani A, Yoshiyama M, Kim S, Omura T, Toda I, Akioka K, et al. Inhibition by angiotensin II type 1 receptor antagonist of cardiac phenotypic modulation after myocardial infarction. J Mol Cell Cardiol. 1995;27(9):1905-14.
9. Humphrey LL, Fu R, Buckley DI, Freeman M, Helfand M. Periodontal disease and coronary heart disease incidence: a systematic review and meta-analysis. J Gen Intern Med. 2008;23(12):2079-86.

10. Sanz M, Marco Del Castillo A, Jepsen S. Periodontitis and cardiovascular diseases: Consensus report. J Gen Intern Med 2020;47(3):268-88.
11. Gonzalez-Navarro B, Pintó-Sala X, Corbella E, Jané-Salas E, Miedema MD, Yeboah J, et al. Associations between self-reported periodontal disease, assessed using a very short questionnaire, cardiovascular disease events and all-cause mortality in a contemporary multi-ethnic population: The Multi-Ethnic Study of Atherosclerosis (MESA). Atherosclerosis. 2018;278:110-6.
