# Supplementary material for: Oral angiotensin-converting enzyme inhibitor captopril protects the heart from Porphyromonas gingivalis LPS-induced cardiac dysfunction in mice
Source: PLoS One. 2023 Nov 20;18(11):e0292624. doi: 10.1371/journal.pone.0292624 (PMC10659197; doi:10.1371/journal.pone.0292624)
Supplement: Attachment — Submitted filename: renamed_90393.pdf [file pone.0292624.s004.pdf]

<u>Additional Editor Comments:</u>

Criticism-1

Please take out the data on AT1A and Bcl2, the corresponding original blots are far from being convincing, since there are much stronger bands than the ones selected proving the non-specificity of the antibodies used.

Response-1:

As requested, we have removed the data on AT_1 receptor and Bcl-2 from the revised manuscript.

Criticism-2:

Also the PKCdelta and PCAMKII blots have a lot of additional bands, and should at least be discussed as possibly misleading.

Response-2:

Indeed, phospho-PKCδ (Tyr-311) blot and phospho-CaMKII (Thr-286) blot have many additional bands, so to select suitable bands for quantification, we carried out chronic infusion of Ang II dissolved in saline for 7 days at a dose of 1,500 ng/kg per min and isolated the heart after 7 days [1, 2]. Control mice received infusion of a comparable volume of saline.

In the phospho-PKCδ (Tyr-311) blot, the density of a single band was increased by Ang II treatment (see **S1A Fig** of **S1 Data)**. In the phospho-CaMKII (Thr-286) blot, the density of two adjacent bands was increased by Ang II treatment (see **S8B Fig** of **S1 Data)**. Therefore, these bands were considered suitable for quantification.

The bands we selected for quantification based on this experiment are consistent with previously reported bands of phospho-PKCδ (Tyr-311) [3] and phospho-CaMKII (Thr-286) [4].

We incorporated the following sentences in the method section of the revised manuscript (Page 12, Line 14-Page 13, Line 3).

Note that the blots of phospho-PKCδ (Tyr-311) and phospho-CaMKII (Thr-286) had many additional bands. Therefore, to identify appropriate bands for quantification, chronic infusion of Ang II (#015-27911; FUJIFILM Wako Pure Chemical Corporation) dissolved in saline was performed for 7 days at a dose of 1,500 ng/kg/min via an osmotic mini-pump (Model 2001; AlZET, Cupertino, CA, USA) and the heart was

isolated after 7 days [1, 2]. Control mice received infusion of a comparable volume of saline (**S1Fig** of **S1 Data)**. Bands whose density was increased in the treated mice were selected for quantification.

We also incorporated the following sentences in the results section of the revised manuscript.

1) Page 18, Lines 13-15
We first confirmed the identify of the band phosphorylated by Ang II, because phospho-PKCδ (Tyr-311) shows many additional bands (**S1A Fig** of **S1 Data)**.

2) Page 20, Lines 15-17
We first confirmed the identify of the bands phosphorylated by Ang II, because phospho-CaMKII (Tyr-286) shows many additional bands (**S1B Fig** of **S1 Data)**.

Reviewer #1:

Criticism-1:

This reviewer recognizes and congratulates the efforts and courage of the authors in returning appropriate answers to the questions initially asked during the reviewing process. Ang II dosage was fundamental to support the conclusions. All researchers who propose western blot technique to detect and quantify proteins, often face antibodies with poor sensitivity and specificity. Although some blots are not satisfactory, it is possible to observe the modulations described in the work, thus being adequate for publication. It is worth warning editors of excellent journals, such as PloS One, in defining the Western blots quality to be accepted for publication so that manufactures will make a great effort to produce antibodies with more sensitivity and specificity.

Resposne-1:

Thank you for your valuable comments.

Reviewer #2:

Criticism-1:

While attempts were made to improve the quality of the data, all immunoblots still are very blurry in the main figures. Most do not match the trend shown in the graphs.

Response-1:

Please refer to our responses to criticism-1 and criticism-2 from the editor. We have removed the data on AT1-AR and Bcl-2 from the revised manuscript as the editor requested. We have also added further explanation regarding multiple bands in the blots.

Criticism-2:

Bcl2 and the PCaMKII blots look overexposed or poor antibody specificity as there are many bands with a stronger signals than the one selected for quantification.

Response-2:

The Bcl-2 blot was removed from the revised manuscript in response to criticism-1 from the editor (please refer to our response to criticism-1 from the editor). Regarding the phospho-CaMKII blot, we have added an explanation of the rationale for band selection (please see our response to criticism-2 from the editor).

Criticism-3:

All immunoblots in the supplement should be labelled in a clear manner so that all bands are labelled for which sample group it belongs to, not just the bands shown in the figure.

Response-3:

We have labelled all immunoblots in the supplemental data as the reviewer required.

References

1. Ren J, Yang M, Qi G, Zheng J, Jia L, Cheng J, et al. Proinflammatory protein CARD9 is essential for infiltration of monocytic fibroblast precursors and cardiac fibrosis caused by angiotensin II infusion. Am J Hypertens. 2011;24(6):701-707.
2. Li Y, Zhang C, Wu Y, Han Y, Cui W, Jia L, et al. Interleukin-12p35 deletion promotes CD4 T-cell-dependent macrophage differentiation and enhances angiotensin II-Induced cardiac fibrosis. Arterioscler Thromb Vasc Biol. 2012;32(7):1662-74.
3. Shan D, Guo S, Wu HK, Lv F, Jin L, Zhang M, et al. Cardiac ischemic preconditioning promotes MG53 secretion through H_2O_2-activated protein kinase C-δ signaling. Circulation. 2020;142(11):1077-1091.
4. Ikeda S, Matsushima S, Okabe K, Ikeda M, Ishikita A, Tadokoro T, et al.

Blockade of L-type Ca^{2+} channel attenuates doxorubicin-induced cardiomyopathy via suppression of CaMKII-NF-κB pathway. Sci Rep. 2019;9(1):9850.
